# Effect of Nourishing “Yin”-Removing “Fire” Chinese Herbal Mixture on Hypothalamic NKB/NK3R Expression in Female Precocious Rats

**DOI:** 10.1155/2014/217424

**Published:** 2014-06-16

**Authors:** Shiran Wang, Liting Zhu, Jian Yu, Zhanzhuang Tian

**Affiliations:** ^1^Department of Neurobiology and Integrative Medicine, Shanghai Medical College, Fudan University, P.O. Box 291, 138 Yi-Xue-Yuan Road, Shanghai 200032, China; ^2^Department of Integrative Medicine, Children's Hospital, Fudan University, 399 WanYuan Road, Shanghai 200032, China

## Abstract

*Aim*. The present study aims to investigate the effects of nourishing “Yin”-removing “Fire” Chinese herb mixture on the hypothalamic NKB/NK3R expression in female precocious model rats. *Materials and Methods*. Female Sprague-Dawley rats were randomly divided into four groups: normal (N), central precocious puberty (CPP) model (M), CPP fed with Chinese herbal mixture (CHM), and CPP fed with normal saline (MS). Rats on postnatal day 5 were given a single subcutaneous injection of 300 *μ*g to establish CPP model rats. Rats of CHM and MS groups were continuously administered with nourishing “Yin”-removing “Fire” Chinese herb mixture or saline since postnatal day 15. The expressions of hypothalamic NKB/NK3R were detected by means of real-time PCR, western blot, and immunofluorescence histochemistry. *Results*. The day of vaginal opening and establishment of two regular estrous cycles were delayed in the CHM group compared with M and MS groups. The expression of hypothalamic NKB/NK3R mRNA and protein in the arcuate nucleus (ARC) and medial preoptic (MPO) area were decreased significantly in the CHM group compared with the M and MS groups on the day of onset-puberty. *Conclusions*. These results indicate that the NKB/NK3R signaling pathway might be involved in the effect of herbal mixture treatment on CPP.

## 1. Introduction 

Precocious puberty is defined by the development of sexual characters before the age of 8 years in girls and 9 years in boys. Idiopathic central precocious puberty (CPP) is the most common type of sexual precocity [1]. It is usually due to the earlier activation of the hypothalamic GnRH neurons. Recently, a lot of studies found that NKB/NK3R signaling pathway plays a critical role in reproduction.

Neurokinin B (NKB) is a 10 amino acid peptide which belongs to the tachykinin family. Three tachykinin receptors are known: NK1, NK2, and NK3. NK3 receptor (NK3R) predominantly mediated the effects of NKB [2]. NKB and NK3R are widely spread throughout the central nervous system. Substantial studies supported that NKB/NK3R regulated GnRH neurons activation in hypothalamus during pubertal period [3]. The expression of* Tac2 *and* Tacr3 *mRNA (encoding NKB and NK3R, resp.) was increased along postnatal maturation in female rat hypothalamus [2]. Several studies found that, similar to GPR54 mutations, human mutations in the TAC and TACR3 (the genes encoding NKB and NK3R, resp.) might result in hypogonadotropic hypogonadism (IHH) [4, 5]. Central administration of NKB agonist (senktide) to rats might induce a profound increase in serum levels of LH, and this effect was regulated by E2-dependent negative feedback [6, 7]. A dramatic increase in LH concentrations was observed after injection of senktide into the third ventricle during the follicular phase in ewes [8]. Single i.v. injection of NKB or senktide elicited robust LH discharges while they were abolished by GnRH receptor antagonism [9]. Recently, neurokinin B (NKB) and dynorphin (Dyn) were found to coexist within kisspeptin neurons in the arcuate nucleus (ARC) where they control the pulsatile release of GnRH [10, 11]. These studies suggest that NKB might be another critical regulator of GnRH release and might involve in the advanced puberty onset in CPP.

GnRH analogue (GnRHa) has been recognized as an effective treatment for advanced puberty onset in CPP patients [12, 13]. Treatment with GnRHa might inhibit the HPG axis of CPP patients, affecting the final height of children, and it would increase the probability of suffering from hyperandrogenism and PCOS when they grow up [13–15]. Traditional Chinese medicines have been successfully used for the management of CPP and improved the clinical symptoms for more than thirty years. The Chinese herb-based formulation nourishing “Yin”-removing “Fire” herbal mixture could downregulate the GnRH expression and significantly delay the sexual development of the precocious puberty rat [16]. It is reported that, Chinese herbal mixture could remarkably reduce the activity of GnRH neurons in the hypothalamus through inhibiting central excitatory amino acid neurotransmitter and promoting central inhibitory amino acid neurotransmitter and beta-endorphin release [17]. We have investigated that the herbal mixture decreased the kiss-1/GPR54 expression in female precocious rats [18]. However, the mechanism of NKB/NK3R signaling participation in the Chinese herb mixture therapeutic effect on precocious puberty is unclear. The present study aims to investigate the effects of nourishing “Yin”-removing “Fire” herbal mixture on hypothalamic NKB/NK3R expression in female precocious rats and further explores the therapeutic mechanism of the herbal mixture in CPP.

## 2. Materials and Methods

### 2.1. Animals and Drugs

Female Sprague-Dawley rats at postnatal day 3 (P3) with their mothers were purchased from Medical Experimental Animals Center of Chinese Academy of Sciences (Shanghai, China). Rats were housed under a 12 : 12 h light/dark cycle with food and water available ad libitum. Animals were randomly divided into normal (N), CPP model (M), CPP model administered with herb mixture (CHM), and CPP model administered with saline (MS) groups. On P5, all CPP model litters were given a single subcutaneous injection of 300 *μ*g of danazol (Hualian Pharm Ltd., Shanghai, China) dissolved in 25 *μ*l vehicle of glycol-ethanol (1 : 1, v/v) [19]. From P15, rats in the CHM and MS were continuously gavaged with nourishing “Yin”-removing “Fire” Chinese herbal mixture or saline 1 mL/50 g body weight, until sacrificed. From P20, rats were inspected daily for vaginal opening (VO); thereafter, vaginal smears were examined daily to identify the period of estrous cycle, until two consecutive regular estrous cycles were established. After two consecutive regular estrous cycles, all animals were sacrificed by decapitation on diestrum estrous cycle with blood and hypothalamus tissue collected. The uterus and ovaries were dissected out and weighed to evaluate the organ coefficients (mg/100 g). The animals used in this study were in accordance with the NIH Guidelines and were approved by Animal Use and Care Committee for Fudan University.

The nourishing “Yin”-removing “Fire” Chinese herbal mixture prescription is mainly composed of 10 medicinal plants: 15 g of* Rehmannia glutinosa* (Sheng di), 9 g each of* Scrophularia buergeriana* (Xuan shen),* Anemarrhena asphodeloides* (Zhi mu), Cortex Phellodendri (Huang bai),* Paeonia suffruticosa* Andr. (Dan pi),* Alisma plantago-aquatica* L. var. orientale Sam. (Ze xie),* Prunella vulgaris* L. (Xia ku cao), 12 g of Carapax et Plastrum Testudinis (Gui jia), 30 g of Fructus hordei germinate (Mai ya), and 6 g of Gentiana scabra Bge (Long Dan Cao). All the above crude drugs were boiling gently in 1000 mL water for 40 min [18]. The mixture was kindly provided by the Department of Integrative Medicine, Children's Hospital of Fudan University, 60 mL/bottle (1 mL containing 3 g of natural medicament power).

### 2.2. Hormone Assay by RIA

The blood samples of all the rats were collected from tail veins, respectively, at the time of sacrifice. The plasma was separated by centrifugation and stored at −80°C until assayed. Concentration of E_2_, LH, and FSH was determined by double-antibody RIA kits purchased from the Beijing Sinouk Institute of Biological Technology (Beijing, China). The samples were assayed in duplicate, and all the subjects' samples were assayed together. The sensitivity of the kit for E2 was less than 5 pg/mL; the intra- and interassay coefficients were less than 10% and 15.2%, respectively. For the sensitivity of the kit for LH, the assay sensitivity was 0.2 mIU/mL, and the intra- and interassay coefficients of variation were 2.0–2.4% and 4.2–7.5%, respectively. For the sensitivity of the kit for FSH, the assay sensitivity was 0.25 mIU/mL, and the intra- and interassay coefficients of variation were 2.2–2.5% and 3.7–8.7%, respectively.

### 2.3. Immunofluorescence Analysis

The effects of nourishing “Yin”-removing “Fire” Chinese herb mixture on the hypothalamic NKB and NK3R expression in different development stages of the rats were investigated. Hypothalamic samples from the four groups were obtained on the day of prepuberty (postnatal 21 days, *n* = 5 per group), onset-puberty (the day of vaginal opening, about postnatal 24 days, *n* = 5 per group), and postpuberty period (establishment of two regular estrous cycles, about postnatal 34 days, *n* = 5 per group). The animals were exsanguinated with normal saline and followed with 4% paraformaldehyde in 0.1 M phosphate buffer (PH 7.4). After perfusion was done, brains were removed and postfixed in 4% paraformaldehyde in 0.1 M PBS (PH 7.4) with 30% sucrose, and their section was sliced at 30 *μ*m thickness on a vibratome microslicer stored at 4°C in tissue culture wells containing 0.1 M PBS (PH 7.4) plus 0.02% sodium azide until further processed.

Slices were treated with 1% Triton X-100 firstly, washed in PBS for 30 min at room temperature (RT), and blocked with 10% normal donkey serum. Then, slices were incubated with a rabbit polyclonal antineurokinin B (NKB, 1 : 500; Novus Biologicals, LLC, USA) or a rabbit polyclonal antineurokinin B receptor (NK3R, 1 : 800; Novus Biologicals, LLC, USA) primary antibodies at 4°C for 24 h. After rinsing, sections were incubated for 1 h at room temperature in the secondary antibody cocktail. For fluorescence detection of the NKB/NK3R antibodies, a 1 : 500 dilution of donkey anti-rabbit Alexa Fluor 488 was used. After secondary labeling, all slices were immediately rinsed and mounted onto subbed slides and coverslipped for analysis by fluorescence microscopy.

### 2.4. Real-Time Reverse Transcriptase-PCR (RT-PCR)

For NKB/NK3RmRNA analysis, the target regions, including mediobasal hypothalamus and the suprachiasmatic-preoptic areas, were dissected on the onset-puberty period. The rat brains of all groups (*n* = 5 per group) were rapidly removed and hypothalamus was separated immediately and frozen in liquid nitrogen. Total hypothalamic RNA was extracted using “TRIzol Reagent” (Invitrogen Inc., America) according to the manufacturer's instructions. The purity and integrity of the RNA were checked spectroscopically and by gel electrophoresis before carrying out the analytical procedures.

The total RNA was digested with RNase-free DNase I (Invitrogen, Carlsbad, CA) before conducting real-time reverse transcriptase-PCR. To obtain cDNA, 2.0 *μ*g of total RNA was reverse transcribed using the SuperScript III reverse transcription system (Invitrogen Corp., Carlsbad, CA, USA) according to the manufacturer's prescriptions.

The primers used for NKB and NK3R mRNA analysis were designed and synthesized by Invitrogen with HPLC purity. Quantitative real-time PCR was carried out by IQ5 real-time PCR detection system (Bio-Rad, Richmond, CA). PCR assay linearity ranges were previously established for each gene cDNA to determine the sensitivity and efficiency of the amplification. The reactions were set up with 11.25 *μ*l SYBR Green RealMasterMix, 1.0 *μ*l primer mixture (200 nM) and 0.5 *μ*l cDNA template. The thermal cycling conditions were as follows: 95°C for 2 min for denaturation, followed by 45 cycles of 95°C for 10 s and 60°C for 20 s, and 72°C for 20 s. After the cycles, a melting curve analysis was performed to insure purity of PCR products.

All real-time experiments were run in triplicate and a mean value was used for the determination of mRNA levels. Relative mRNA expression levels for NKB and NK3R were analyzed using the formula 2^−ΔΔCt^ method and normalized to the *β*-actin ribosomal RNA.

### 2.5. Western Blot Analyses

On the day of onset-puberty in M, all rats of four groups were sacrificed by decapitation. NK3R protein expression was investigated by western blot with a standard procedure. After rapid removal of the brain, the hypothalamus has been removed, snapped frozen in liquid nitrogen, and stored at −80°C. For total protein extraction, hypothalamus was homogenized in 300 *μ*l RIPA lysis Buffer (Beyotime, China) with protease inhibitors (phenylmethanesulfonyl fluoride, Beyotime, China) using a Polytron homogenizer. Tissue lysates were cleared by centrifugation at 13,000 rpm for 15 min at 4°C. Protein content was determined by the BCA method (Bio-Rad). Samples were boiled with loading buffer (1650 mM Tris-HCl, 2% SDS, 10% glycerol, 10% *β*-mercaptoethanol, and 0.001% bromophenol blue) for 10 min and stored at −80°C until use.

Protein aliquots were separated on 10% SDS polyacrylamide gels for 70 min at 110 V. Then proteins were transferred onto polyvinyldifluoride membranes (Millipore, USA) in a Trans-Blot apparatus (Bio-Rad Laboratories, Inc.) for 110 min at 100 V. Membranes were blocked for 2 h in blocking buffer (TBS with 0.1% Tween 20 (TBST) and 5% BSA) at RT. The membranes were then incubated overnight at 4°C with primary antibody (rabbit polyclonal antineurokinin B receptor (NK3R, 1 : 500; Abbiotec)) diluted in the same buffer solution containing BSA described above. After washing the membranes extensively in washing buffer (TBS-0.1% Tween 20), the membrane was incubated with HRP-conjugated donkey anti-rabbit IgG (1 : 10000, Millipore) diluted in blocking buffer for 2 h at 4°C. The membrane was washed several times in the washing buffer to remove unbound secondary antibody and the signal was detected by ECL detection kit (GE Healthcare). The membranes were exposed in Image Quant LAS 4000 mini (GE Healthcare), and signals were determined by ImageJ software. The results were expressed according to the intensity of the signals in arbitrary densitometric units after normalization by glyceraldehyde-3-phosphate dehydrogenase (GAPDH, Sigma, USA) as an internal standard.

### 2.6. Statistical Analysis

Values were expressed as mean ± SEM. Statistical analysis was performed on raw data using the one-way ANOVA, with the significance concentrations of *P* < 0.05 in two-tailed testing chosen. Comparisons among groups were made using the Student's *t*-test.

## 3. Results

### 3.1. The Day of Vaginal Opening and the Establishment of Two Regular Estrous Cycles of the Rats

**Figure 1:**
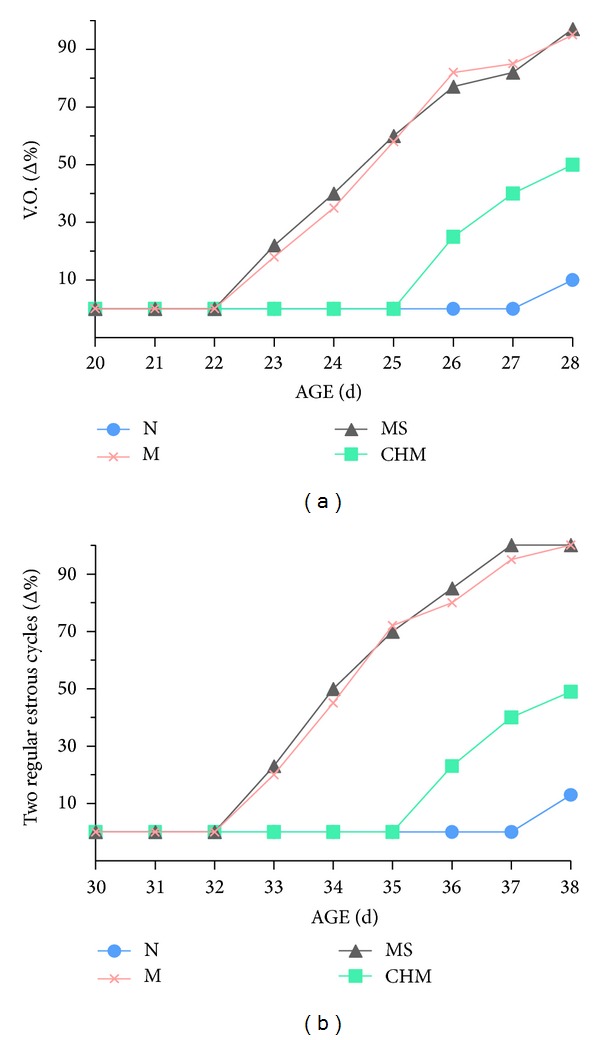
Effects of Chinese herb mixture on the day of vaginal opening and establishment of two regular estrous cyclesof female precocious rats. The day of vaginal opening and establishment of two regular estrous cycles were earlier in M and MS than N and then were delayed by administration of Chinese herb mixture. N: normal, M: model, MS: saline, and CHM: Chinese herb mixture.

The day of vaginal opening and the establishment of two regular estrous cycles were significantly advanced in M than that in N (*P* < 0.01, Figure 1). The day of vaginal opening and the establishment of two regular estrous cycles in CHM were significantly delayed than those of M and MS (*P* < 0.01, Figure 1).

### 3.2. Organ Coefficients of Ovary and Uterus

**Figure 2:**
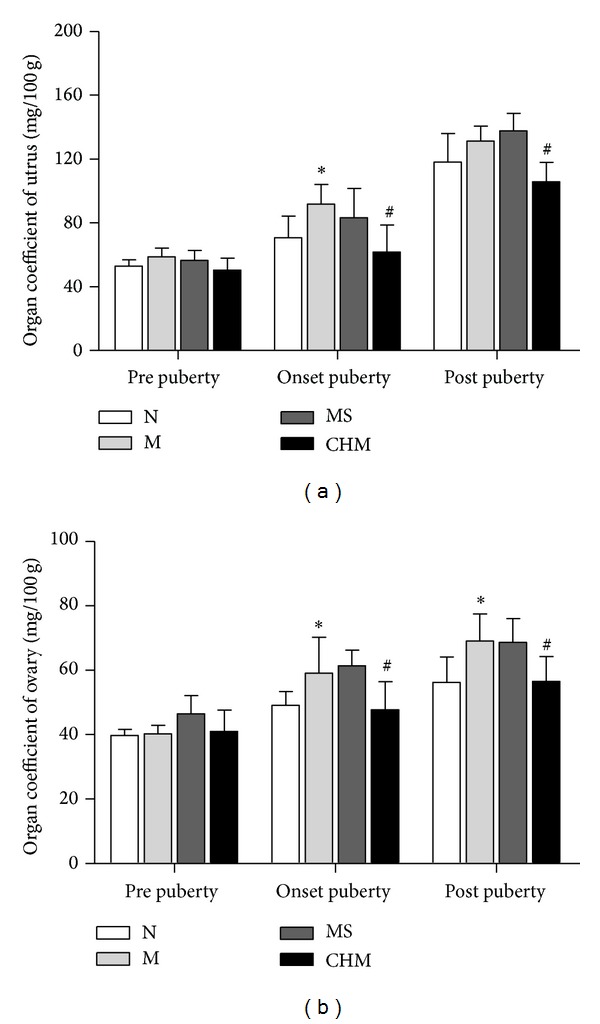
Effects of Chinese herb mixture on uterus and ovary coefficients of female precocious rats. On prepuberty, there were no significant differences among four groups; the ovary and uterus coefficients of M have significant predominance over N groups (*P* < 0.05), and CHM groups have decreased the organ coefficients in M of onset-puberty (*P* < 0.05) and postpuberty (*P* < 0.05). N: normal, M: model, MS: saline, and CHM: Chinese herb mixture. *P < 0.05 versus N; ^#^ *P* < 0.05 versus M.

On the day of prepuberty, the organ coefficients of uteri and ovaries have no significant differences among four groups. On the day of onset-puberty, the uteri and ovaries coefficients of the M were increased more significantly than those of N (*P* < 0.05, *P* < 0.05, resp.), while they were decreased significantly in CHM compared with those of M (*P* < 0.05, *P* < 0.05, resp.) and MS; On the day of postpuberty, the ovaries coefficient of the M was significantly upregulated compared with that of N (*P* < 0.05); the organ coefficients of uteri and ovaries in CHM were decreased obviously compared with those of M (*P* < 0.05, *P* < 0.05, resp.) and MS (Figure 2).

### 3.3. Hormone Levels in Blood of the Rats

Plasma E_2_, FSH levels in M were higher than those in N (*P* < 0.05, *P* < 0.05, resp.), while, the plasma E_2_, FSH levels in CHM were significantly decreased compared with those in M (*P* < 0.05, *P* < 0.05, resp.) and MS. However there were no significant differences in plasma LH levels among four groups (Table 1).

### 3.4. Effects of Chinese Herbal Mixture on Hypothalamic NKB Expression by Immunofluorescence

There were few NKBir cells found in the hypothalamus in N, M, MS, and CHM on the day of prepuberty.

On the day of onset-puberty, the number of NKBir cells in ARC of M was obviously increased compared with those in N (*P* < 0.01). The number of positive NKBir cells in ARC was significantly decreased in CHM compared with those in M (*P* < 0.05). There was no statistical difference between M and MS.

On the day of onset-puberty, the number of NKBir cells in MPO of M was obviously increased compared with those in N (*P* < 0.05). The number of positive NKBir cells in MPO was significantly decreased in the CHM compared with those in M (*P* < 0.05). There was no statistical difference between M and MS.

**Figure 3:**
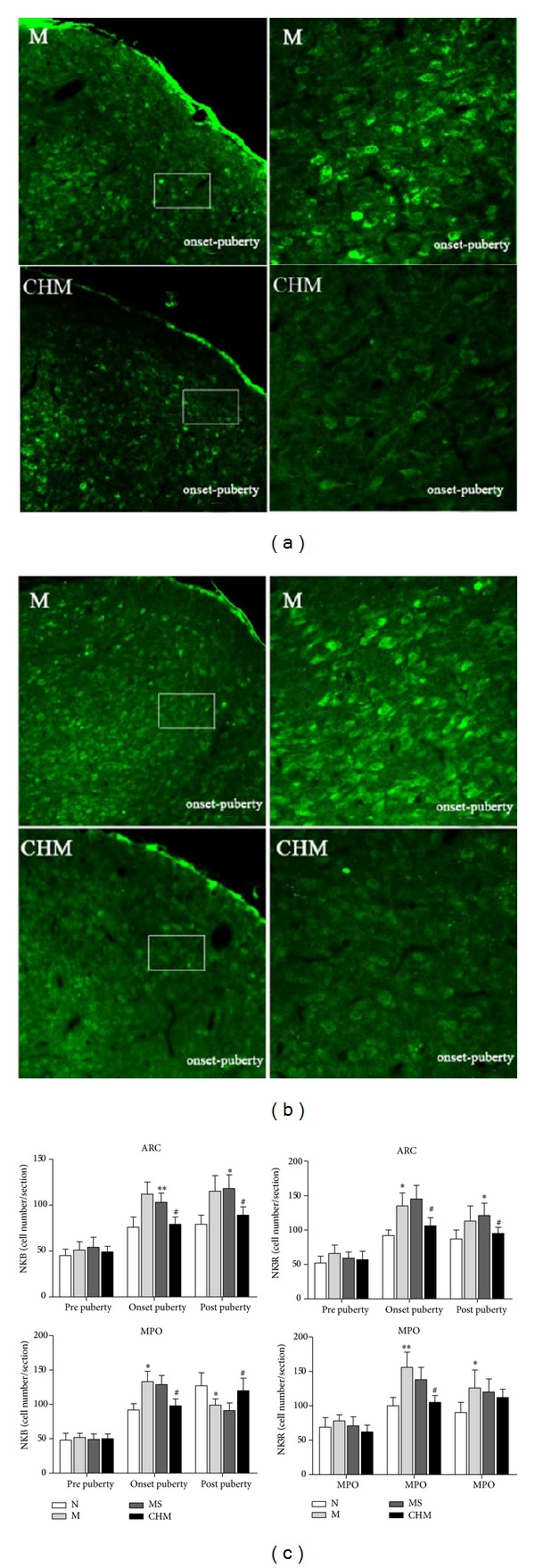
Effect of Chinese herb mixture on hypothalamic NKB/NK3R expression by immunofluorescence. (a) Representative microscopic images showing that the NKBir positive neurons located in ARC on the day of onset-puberty decreased in CHM rats compared with M ones (low magnification 10x, high magnification 40x). (b) Representative microscopic images showing that the NK3Rir positive neurons located in ARC on the day of onset-puberty decreased in CHM rats compared to M (low magnification 10x, high magnification 40x). (c) Calculated number of the NKB and NK3R neurons in the ARC and MPO in all four groups of rats (*n* = 5 per group). All observations from individual animal were averaged for that animal, and then these single numbers of each animal were used to calculate the group mean. The data is expressed as the mean with SEM. N and MS groups were not shown in the figure. N: normal, M: model, MS: saline, and CHM: Chinese herb mixture. *P < 0.05 versus N; ^#^ *P* < 0.05 versus M; **P < 0.01 versus N.

On the day of postpuberty, the NKBir cell numbers in ARC were significantly higher in M than those in N (*P* < 0.05), and they decreased in CHM than those in M and MS (*P* < 0.05). In MPO, the number of NKBir cells in M was less than that in N (*P* < 0.05), and it increased in the CHM (*P* < 0.05) than that in M (Figure 3).

### 3.5. Effects of Chinese Herbal Mixture on Hypothalamic NK3R Expression by Immunofluorescence

There were few NK3Rir cells found in the hypothalamus in N, M, MS, and CHM during the prepuberty period.

On the day of onset-puberty, the number of NK3Rir cells in ARC of M was obviously increased compared with that in N (*P* < 0.05). The number of positive NK3Rir cells in ARC was significantly decreased in the CHM compared with that in M (*P* < 0.05). There was no statistical difference between M and MS.

On the day of onset-puberty, the number of NK3Rir cells in MPO of M was obviously increased compared with that in N (*P* < 0.01). The number of positive NK3Rir cells in MPO was significantly decreased in the CHM compared with that in M (*P* < 0.05). There was no statistical difference between M and MS.

On the day of postpuberty, the NK3Rir cell numbers in ARC were significantly higher in M than those in N (*P* < 0.05) and decreased in CHM than those in M and MS (*P* < 0.05). The NK3Rir cell numbers in MPO were higher in M than in N (*P* < 0.05), and there was no difference between CHM and M (Figure 3).

### 3.6. Effects of Chinese Herbal Mixture on the Expression of NK3R by Western Blot

**Figure 4:**
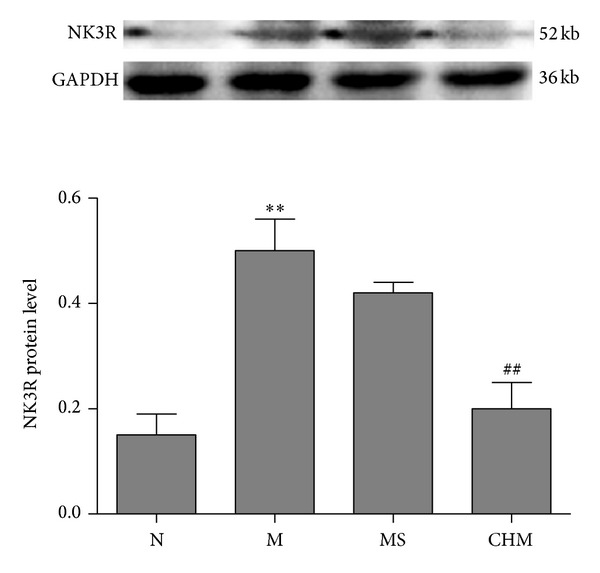
Effects of Chinese herb mixture on hypothalamus NK3R protein expression by western blot. The expression of NK3R protein was upregulated in M rats compared to N (*P* < 0.01) and downregulated under the influence of Chinese herb mixture (*P* < 0.01). *n* = 5 per group. N: normal, M: model, MS: saline, and CHM: Chinese herb mixture. **P < 0.01 versus N; ^##^ *P* < 0.01 versus M.

Effect of Chinese herb mixture on expression of NK3R was also detected by means of western blot. The hypothalamus NK3R expression increased significantly in M compared with that of N (*P* < 0.01) and decreased in CHM compared with that in M (*P* < 0.01). There were no statistical difference between M and MS (Figure 4).

### 3.7. Effects of Chinese Herbal Mixture on Hypothalamic NKB mRNA Expression by Real-Time PCR

**Figure 5:**
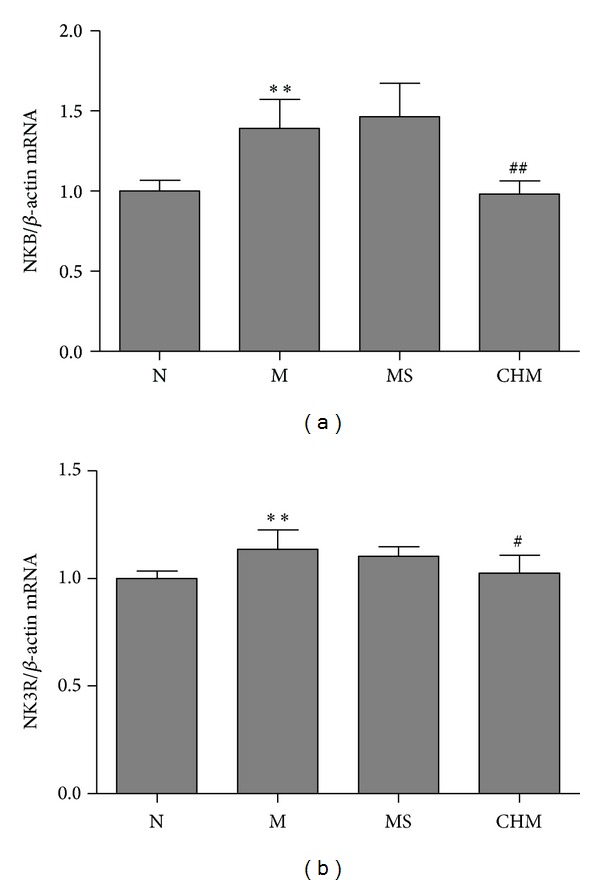
Effects of Chinese herb mixture on hypothalamic NKB/NK3R mRNA expression by real-time PCR. (a) The expression of NKB mRNA in M has significantly increased compared to N (*P* < 0.01) and decreased in CHM compared to M (*P* < 0.01). (b) The picture shows that the expression of NK3R mRNA in M was higher than in N (*P* < 0.01), and that Chinese herb mixture was downregulated NK3R mRNA expression compared to M (*P* < 0.05). Results analysis used the ratio of NKB or NK3R/*β*-actin (*n* = 5 per group) expressed as the mean with SEM. N: normal, M: model, MS: saline, and CHM: Chinese herb mixture. **P < 0.01 versus N; ^#^ *P* < 0.05 versus M; ^##^ *P* < 0.01 versus M.

Relative mRNA levels for NKB were detected by real-time reverse transcriptase-PCR when puberty onset. Analysis of the NK3R mRNA concentration by the 2^−ΔΔCt^ method and normalized to the *β*-actin mRNA expressed as the mean with SEM. The NKB mRNA in the M increased significantly compared with that in the N (*P* < 0.01), while NKB mRNA in the CHM decreased significantly compared with that in the M (*P* < 0.01) (Figure 5).

### 3.8. Effects of Chinese Herbal Mixture on Hypothalamic NK3R mRNA Expression by Real-Time PCR

The* expression* of* hypothalamic *NK3R mRNA at onset-puberty was detected by real-time reverse transcriptase-PCR. We used the 2^−ΔΔCt^ method to analysis the data, and then normalized to *β*-actin. The ratio of NK3R to *β*-actin in M was significantly elevated compared to that in N (*P* < 0.01), and it was downregulated in CHM compared to that of M (*P* < 0.05). There was no statistical difference between M and MS (Figure 5).

## 4. Discussion

Pubertal development occuring before the age of 8 years in girls and the age of 9 years in boys is defined as precocious puberty. Precocious puberty has a profound impact on growth, development, and psychosocial well-being of the patients [1, 12]. Chinese herbal mixture was proved efficient in modulating the course of puberty development [16]. But the precise mechanism of the herbal mixture is still unclear. NKB/NK3R signaling pathway was found to play a critical role in puberty development. The present study is to investigate the influence of nourishing “Yin”-removing “Fire” Chinese herbal mixture on NKB/NK3R expression in precocious puberty.

Our experiment showed that the day of vaginal opening and the establishment of two regular cycles were obviously delayed, after administration of CPP model rats with nourishing “Yin”-removing “Fire” Chinese herbal mixture. On the day of onset-puberty, the serum levels of E2 and FSH in CHM were also significantly reduced by Chinese herbal mixture. The Chinese herbal mixture could postpone the development of uterine and ovarian in CPP rats. To further explore the role of NKB and NK3R in precocious puberty, the expression of NKB and NK3R in the arcuate nucleus (ARC) and medial preoptic (MPO) was observed at different stages of puberty. We found that the number of NKB/NK3R positive cells in hypothalamus of M was significantly increased upon puberty onset; the expression of NKB/NK3R mRNA and NK3R protein was also upregulated on the day of onset-puberty, indicating that the NKB/NK3R signaling might be involved in the activation of puberty and advance of puberty. The increased NKB cell number might play an exciting role on GnRH secretion by projecting to the GnRH neurons at ME [3] upon puberty onset. On the day of establishment of two regular estrous cycles, the expression trends of NKB/NK3R in ARC were consistent with the day of onset-puberty; probably for that the ARC maintained a basic secretion of GnRH, which did not change significantly during the estrus cycle. In MPO, the number of NKBir cells was significantly decreased in M compared to that of N on postpuberty, maybe because the MPO area mainly maintained the cyclical secretion of GnRH, and was strongly influenced by serum estrogen level [20, 21]. ARC and MPO areas were important in the modulation of hypothalamus neuroendocrine function and were closely related to the regulation of GnRH neurons [7, 22]. The different expressions of NKB/NK3R in ARC and MPO indicated that hypothalamic ARC and MPO played different roles in promoting puberty initiate and normal sexual cycles and finally maintained the normal development of puberty together.

After giving precocious puberty rats with nourishing “Yin”-removing “Fire” Chinese herbal mixture, the number of NKB/NK3Rir neurons in ARC and MPO was decreased significantly on the day of onset-puberty; the NKB/NK3R mRNA and NK3R protein expression in hypothalamus was also reduced significantly, which showed that the nourishing “Yin”-removing “Fire” Chinese herbal mixture not only reduced the hypothalamus TAC3 and TAC3R transcription during precocious rats, downregulated NKB and NK3R mRNA synthesis, but also inhibited the release of NKB and expression of NK3R. NKB neurons have direct connection with GnRH neurons and fibers [3], and there are NK3R expression on GnRH neurons [6], we speculated that the nourishing “Yin”-removing “Fire” Chinese herbal mixture may influence the GnRH release through inhibiting NKB and NK3R expression and then delay the HPGA initiation. The role of NKB involved in the therapeutic effects of Chinese herbal mixture on precocious puberty rats is implemented by its receptor NK3R.

## 5. Conclusion

NKB/NK3R signaling might be involved in the advance onset-puberty in precocious puberty and the regulation of nourishing “Yin”-removing “Fire” Chinese herb mixture on the abnormal function of HPGA in precocious puberty rats. The potential mechanism of the herb mixture needs to be further revealed so as to bring evidence to clinical exercise.

## Figures and Tables

**Table 1:** Effects of Chinese herb mixture on serum E_2_, FSH and LH levels of female precocious rats.

	E_2_ (pg/mL)	FSH (mIU/mL)	LH (mIU/mL)
N	29.14 ± 3.63	15.58 ± 1.11	16.16 ± 0.68
M	35.93 ± 5.45*	18.1 ± 3.28*	17 ± 0.98
MS	35.05 ± 2.65	17.16 ± 3.83	17.34 ± 0.79
CHM	32.15 ± 5.72^#^	15.59 ± 1.37^#^	16.89 ± 0.86

The serum level of E_2_ (*P* < 0.05) and FSH (*P* < 0.05) significantly increased in M compared with N, and then the level of E_2_ (*P* < 0.05) and FSH (*P* < 0.05) was decreased in CHM. There were no significant differences between M and MS. N: normal, M: model, MS: saline, and CHM: Chinese herb mixture.

*P < 0.05 versus N; ^#^ *P* < 0.05 versus M.
